# Low Ozone Concentrations Differentially Affect the Structural and Functional Features of Non-Activated and Activated Fibroblasts In Vitro

**DOI:** 10.3390/ijms221810133

**Published:** 2021-09-20

**Authors:** Barbara Cisterna, Manuela Costanzo, Maria Assunta Lacavalla, Mirco Galiè, Osvaldo Angelini, Gabriele Tabaracci, Manuela Malatesta

**Affiliations:** 1 Department of Neurosciences, Biomedicine and Movement Sciences, Anatomy and Histology Section, University of Verona, Strada Le Grazie 8, I-37134 Verona, Italy; barbara.cisterna@univr.it (B.C.); manuela.costanzo@univr.it (M.C.); mariaassunta.lacavalla@univr.it (M.A.L.); mirco.galie@univr.it (M.G.); 2 San Rocco Clinic, Via Monsignor G.V. Moreni 95, I-25018 Montichari, Italy; osva.ange@virgilio.it (O.A.); tabaracci@sanrocco.net (G.T.)

**Keywords:** oxygen–ozone therapy, cell proliferation, cell surface protrusions, nuclear factor erythroid 2-related factor 2 (Nrf2), heme oxygenase 1 (Hmox1) gene, interleukin-6, transforming growth factor (TGF)-β1, fluorescence microscopy, scanning electron microscopy, real-time quantitative polymerase chain reaction (RT-qPCR)

## Abstract

Oxygen–ozone (O_2_–O_3_) therapy is increasingly applied as a complementary/adjuvant treatment for several diseases; however, the biological mechanisms accounting for the efficacy of low O_3_ concentrations need further investigations to understand the possibly multiple effects on the different cell types. In this work, we focused our attention on fibroblasts as ubiquitous connective cells playing roles in the body architecture, in the homeostasis of tissue-resident cells, and in many physiological and pathological processes. Using an established human fibroblast cell line as an in vitro model, we adopted a multimodal approach to explore a panel of cell structural and functional features, combining light and electron microscopy, Western blot analysis, real-time quantitative polymerase chain reaction, and multiplex assays for cytokines. The administration of O_2_–O_3_ gas mixtures induced multiple effects on fibroblasts, depending on their activation state: in non-activated fibroblasts, O_3_ stimulated proliferation, formation of cell surface protrusions, antioxidant response, and IL-6 and TGF-β1 secretion, while in LPS-activated fibroblasts, O_3_ stimulated only antioxidant response and cytokines secretion. Therefore, the low O_3_ concentrations used in this study induced activation-like responses in non-activated fibroblasts, whereas in already activated fibroblasts, the cell protective capability was potentiated.

## 1. Introduction

In the last decades, the medical use of gaseous ozone (O_3_) has been progressively increasing as a complementary/adjuvant treatment for several diseases [1,2,3,4]. O_3_ is a highly unstable gas rapidly decomposing to oxygen, and it is applied for therapeutic purposes as O_2_–O_3_ mixtures with low O_3_ concentrations. In fact, the mild oxidative stress induced by low doses of O_3_ activates the nuclear factor erythroid 2-related factor 2 (Nrf2)-mediated Keap1-dependent pathway, which, in turn, stimulates gene expression of antioxidant response elements (ARE) [5,6,7]. In fact, the exposure to low O_3_ concentrations promotes an antioxidant cytoprotective response [8,9], which is consistent with the principle of hormesis, i.e., “the beneficial effect of a low-level exposure to an agent that is harmful at high levels” [10].

Despite the wide application of O_2_–O_3_ administration in clinical practice, the biological mechanisms accounting for the therapeutic efficacy of O_3_ have been only partially unveiled, and further investigations are necessary to understand the multiple effects of this gas on the different cell types in tissues and organs. As an example, appropriate O_2_–O_3_ gas mixtures proved to exert an adipogenic effect in human adipose-derived adult stem cells [11] and to reduce lipid loss in explanted adipose tissue [6]. In addition, it has recently been demonstrated that, besides activating an antioxidant response through the Nrf2–ARE pathway, O_2_–O_3_ gas mixtures are able to modulate cytokine secretion in T lymphocytes [7].

In the present work, we focused our attention on the fibroblast as a primary target of O_2_–O_3_ therapy. In fact, it is worth noting that, whatever the administration route (intramuscular, intra- and peri-articular or subcutaneous injection, topical bagging, rectal or vaginal insufflation, autohemotherapy), O_2_–O_3_ mixtures inevitably act on fibroblasts; actually, these are ubiquitous cells playing multiple roles in the architecture of organs and the whole body, in the homeostasis of tissue-resident cells, and in many physiological (e.g., wound healing) and pathological processes, such as autoimmunity, fibrosis, and cancer [12]. It is therefore likely to expect that fibroblasts may be involved in the pathways activated by O_2_–O_3_ administration and, in turn, in the therapeutic outcome.

An established human fibroblast cell line was used as a suitable in vitro model to study the response to O_2_–O_3_ treatment under strictly controlled experimental conditions. We investigated the structural and functional effects of low O_3_ concentrations on fibroblasts in non-activated and lipopolysaccharide (LPS)-activated state with the aim to analyze the response of these tissue-resident cells in a steady state or after the response to activating stimuli (as it happens following tissue injury and/or inflammation, when they are committed to restore homeostasis [13]). We used gas mixtures with 10, 20, and 30 μg O_3_/mL O_2_—concentrations usually applied in clinical practice. In order to explore a panel of structural and functional cell features, a multimodal approach was adopted, by combining light (bright field and fluorescence) microscopy, scanning electron microscopy (SEM), Western blot analysis, real-time quantitative polymerase chain reaction (RT-qPCR), and multiplex assays for cytokines.

## 2. Results

### 2.1. Cytotoxicity

**Figure 1:**
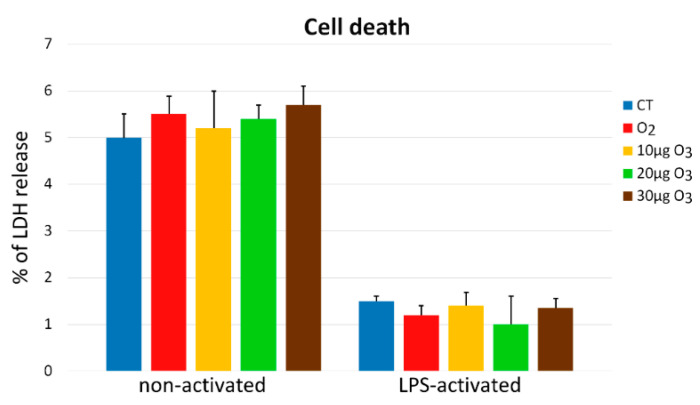
Percentages of dead cells (mean values ± SE) 24 h after the treatment (one experiment in triplicate). No significant difference was found among the samples of either group. CT—untreated control.

At 24 h after the gas exposure, the percentage of dead cells (estimated by the LDH release) was similar (<6%) in all samples of non-activated fibroblasts, irrespective of the treatment (*p* = 0.77, Figure 1). Similarly, no difference in cell death was found among the LPS-activated samples (*p* = 0.11; Figure 1). LPS-activated control samples showed a significantly lower value (<2%; *p* = 0.02) in comparison with the non-activated controls.

### 2.2. S-Phase Evaluation

**Figure 2:**
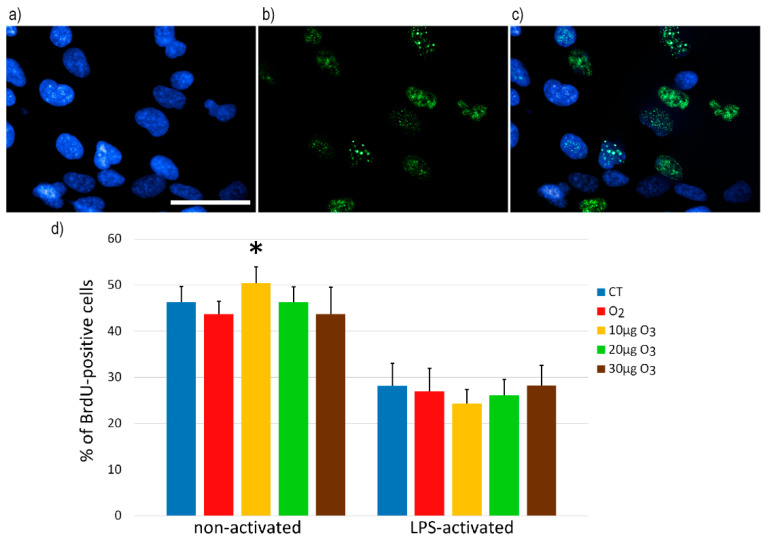
(**a**–**c**) Representative fluorescence microscopy images of fibroblasts stained for DNA with Hoechst 33342 (blue) (**a**), immunolabelled for BrdU (green) (**b**), and merged (**c**). Bar, 100 μm. (**d**) Mean values ± SE of percentages of BrdU-positive cells 24 h after the treatment (one experiment in triplicate). The asterisk (*) indicates significant difference in comparison with the respective control (CT).

Under a conventional fluorescence microscope, bromodeoxyuridine (BrdU)-positive fibroblasts appeared labelled in green, while DNA was counterstained in blue (Figure 2a–c). At 24 h after the gas treatment, significant difference in the percentage of BrdU-positive cells was found in non-activated fibroblasts (*p* = 0.01); post-hoc analysis revealed that only cells exposed to 10 μg O_3_ showed a significantly increased proliferation rate in comparison with the controls (*p* = 0.04) (Figure 2d). In LPS-activated fibroblasts, the exposure to O_2_ or O_3_ did not alter the percentage of BrdU-positive cells (*p* = 0.52) (Figure 2d). LPS-activated control samples showed a significantly lower cell proliferation in comparison with the non-activated controls (*p* < 0.001).

### 2.3. Wound Healing Assay

**Figure 3:**
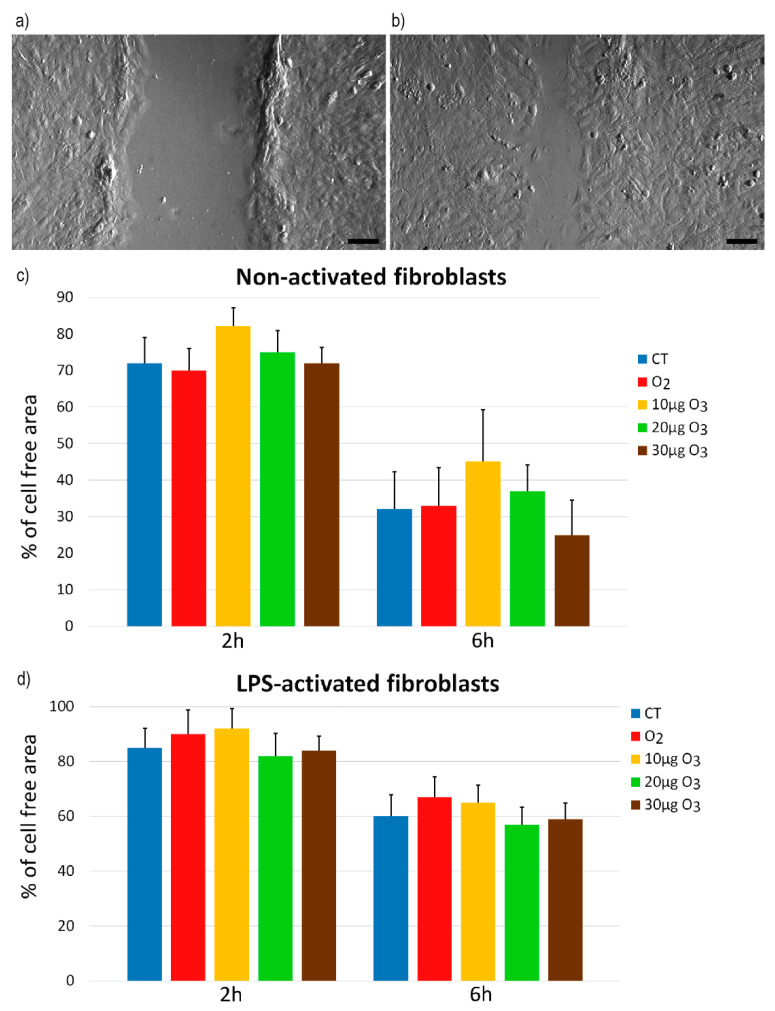
(**a**,**b**) Representative inverted microscope images of fibroblasts at 2 h (**a**) and 6 h (**b**) of the wound healing assay. Bars, 200 µm. (**c**,**d**) Means ± SE of percentages of cell-free areas of control (CT), O_2_- and O_3_-treated non-activated (**c**) and LPS-activated (**d**) fibroblasts at 2 h and 6 h of the wound healing assay (three experiments). No statistical difference was found for both non-activated and LPS-activated samples.

The wound healing assay (representative images in Figure 3a,b) showed similar migration rates in all samples of both non-activated (Figure S1) and LPS-activated (Figure S2) fibroblasts 2 h (*p* = 0.21 and *p* = 0.33, respectively) and 6 h (*p* = 0.06 and *p* = 0.76, respectively) (Figure 3c,d) after gas exposure. After 24 h, the wound was completely healed in all samples (not shown).

### 2.4. Scanning Electron Microscopy

**Figure 4:**
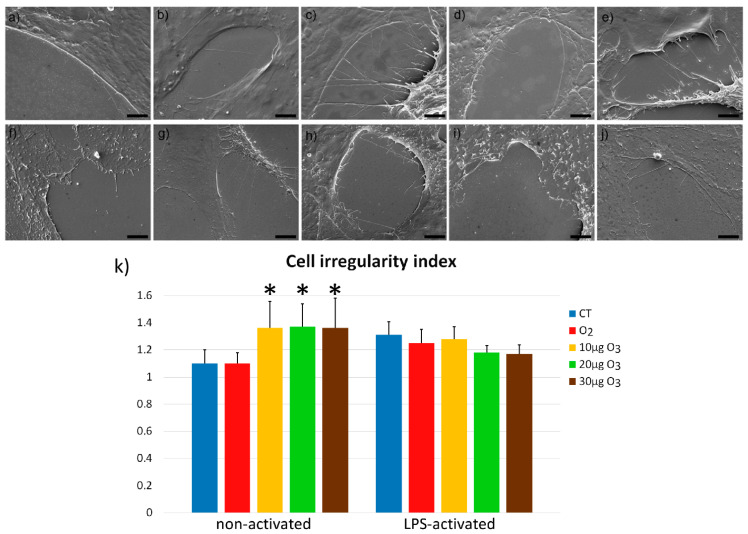
Scanning electron micrographs of non-activated (**a**–**e**) and LPS-activated (**f**–**j**) fibroblasts in control (**a**,**f**) and 24 h after exposure to O_2_ (**b**,**g**), 10 μg O_3_ (**c**,**h**), 20 μg O_3_ (**d**,**i**), and 30 μg O_3_ (**e**,**j**). Bars, 10 μm. (**k**) Means ± SE of the cell irregularity index at 24 h after the treatment in non-activated or LPS-activated fibroblasts (one experiment). Significant increase in the cell irregularity index was found in non-activated fibroblasts after O_3_ treatments (*).

In non-activated condition, control and O_2_-treated fibroblasts appeared flattened and irregularly polygonal in shape, with scarce filamentous protrusions of the cell surface (Figure 4a,b). O_3_-treated fibroblasts maintained the polygonal shape but showed an increase in the surface projections (Figure 4c–e). In LPS-activated condition, all samples showed evident surface protrusions (Figure 4f–j).

Quantitative evaluation of the surface irregularities showed significant differences in non-activated fibroblasts (*p* = 0.006), confirming that the protrusions of O_3_-treated samples significantly increased (*p* < 0.01) in comparison with control and O_2_-treated samples (Figure 4k). No significant difference in surface irregularity was found among LPS-activated samples (*p* = 0.09) (Figure 4k). 

In LPS-activated condition, control samples had a significant increase in surface protrusions in comparison with the non-activated controls (*p* = 0.03).

### 2.5. Western Blot Analysis

**Figure 5:**
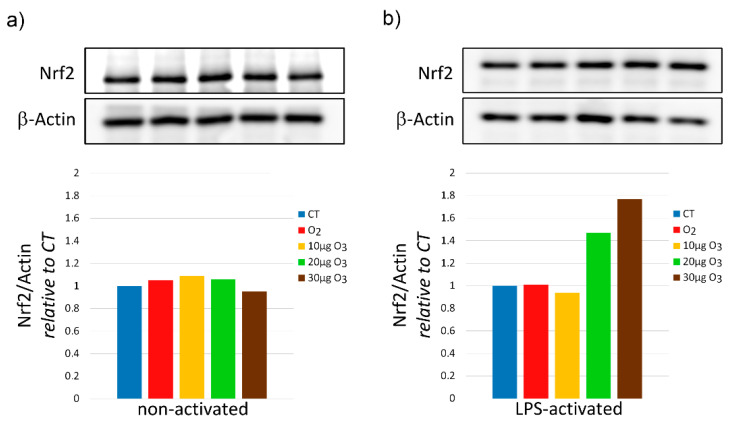
Western blot of Nrf2 protein at 20 min after treatment of non- activated (**a**) and activated (**b**) fibroblasts (one experiment in duplicate). Data were normalized to the level of a housekeeping protein (actin) and expressed as in proportion to the levels in control (CT) sample.

In non-activated fibroblasts, the total amount of Nrf2 protein was similar in all samples (Figure 5a). In LPS-activated fibroblasts, the Nrf2 protein content showed an evident increase in the samples treated with 20 μg O_3_ and 30 μg O_3_ in comparison with the control (Figure 5b).

### 2.6. Real-Time Quantitative Polymerase Chain Reaction

**Figure 6:**
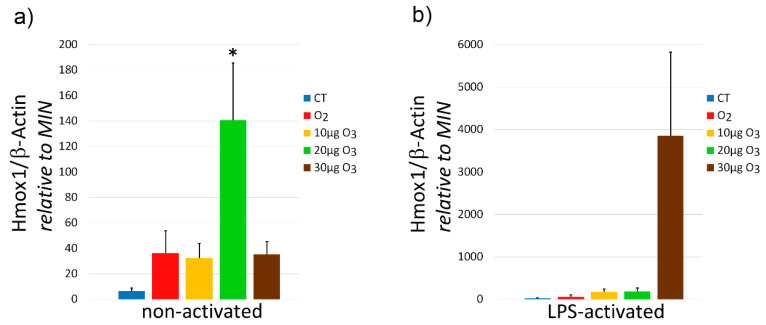
Hmox1 gene expression (means ± SE) in non-activated (**a**) and LPS-activated (**b**) fibroblasts at 24 h after treatment (one experiment in triplicate). Asterisk (*) indicates significant difference with control (CT).

In non-activated fibroblasts, the one-way ANOVA test demonstrated significantly different Heme oxygenase 1 (Hmox1) gene expression (*p* = 0.03) among samples; in particular, 20 μg O_3_-treated fibroblasts showed values significantly higher in comparison with control (*p* = 0.002) (Figure 6a). On the other hand, no significant linear trend was found (*p* = 0.09). In LPS-activated fibroblasts, the one-way ANOVA test showed no significant difference (*p* = 0.07) among samples (Figure 6b), but the test for linear trend demonstrated a dose-dependent trend (*p* = 0.03).

### 2.7. Interleukin-6 and Transforming Growth Factor-β1 Secretion

**Figure 7:**
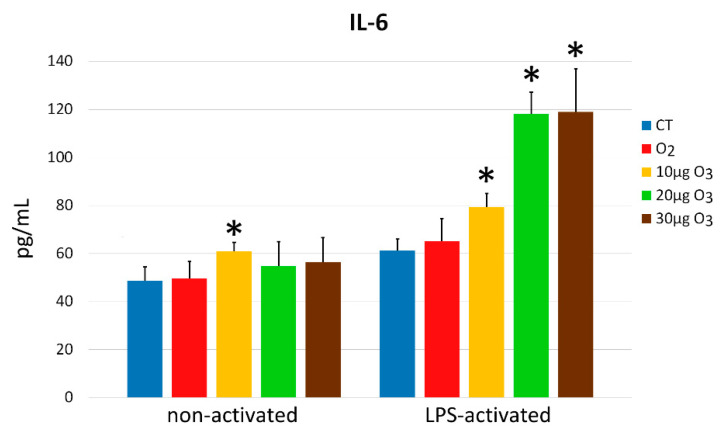
IL-6 amounts (mean values ± SE) detected in the medium of non-activated and LPS-activated cell samples 24 h after gas treatment (two experiments in duplicate). Asterisks (*) indicate significant differences from the respective controls (CT).

In the non-activated condition, a significant difference was found in the amount of interleukin (IL)-6 secreted into the medium (*p* = 0.03); in particular, a significantly higher value was found in 10 μg O_3_-treated samples in comparison with all other samples (*p* < 0.05) (Figure 7). Significant difference was found also in the LPS-activated state (*p* = 0.004), where O_3_-treated samples secreted a significantly higher amount of IL-6 in comparison with control and O_2_-treated samples (*p* < 0.05). 

Moreover, LPS-activated control fibroblasts secreted significantly higher IL-6 amounts in comparison with non-activated control samples (*p* = 0.02) (Figure 7). 

**Figure 8:**
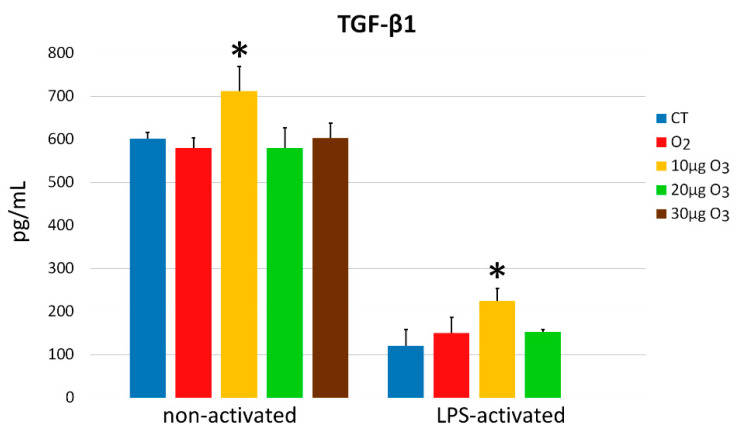
TGF-β1 amounts (mean values ± SE) detected in the medium of non-activated and LPS-activated cell samples 24 h after gas treatment (two experiments in duplicate). In LPS-activated condition, the value of 30 μg O_3_-treated samples was below the detection limit. Asterisks (*) indicate significant differences from the respective controls (CT).

In the non-activated condition, a significant difference was found in the amount of transforming growth factor (TGF)-β1 secreted into the medium (*p* = 0.01); in detail, a significantly higher value was found in 10 μg O_3_-treated samples in comparison with all other samples (*p* < 0.05) (Figure 8). Similarly, significant difference was found in LPS-activated fibroblasts (*p* < 0.001), where the treatment with 10 μg O_3_ induced a significant increase in secreted TGF-β1 in comparison with the other samples (*p* < 0.05), while the value in 30 μg O_3_-treated samples was below the detection limit. 

In addition, LPS-activated fibroblasts secreted significantly lower amounts of TGF-β1 than non-activated control samples (*p* = 0.03) (Figure 8).

## 3. Discussion

In the present investigation, we evaluated the effects of the exposure to O_2_–O_3_ mixtures at low O_3_ concentrations on the structural and functional features of fibroblasts as a cell type ubiquitously distributed in body tissues. Being primarily responsible for the deposition and degradation of the extracellular matrix, fibroblasts play a key role in tissue remodeling [14] and wound healing [12] and are also involved in the immune response and, more generally, in the maintenance of tissue homeostasis [13]. The molecular and cellular effects of low O_3_ concentrations were investigated in both non-activated and LPS-activated fibroblasts, with the aim of getting information on the possibly differential response due to the cell functional state.

LDH assay showed that the exposure to any of the gas mixtures used did not induce significant cytotoxicity compared with the controls, in both non-activated and LPS-activated fibroblasts, consistent with previous data on other cell types [6,7,11,15,16]. The LDH values were lower for the LPS-treated than for the non-activated samples: this is likely due to the activated state itself, as it has been demonstrated that pro-survival mechanisms are stimulated in activated fibroblasts when these cells are committed to tissue repair [17].

Based on the evaluation of BrdU-positive S-phase cells, non-activated fibroblasts proved to be stimulated by the exposure to 10 μg O_3_, suggesting that the eustress induced by this mild concentration may promote cell growth, which is especially advantageous in the post-injury tissue repair [18,19]. Accordingly, non-activated fibroblasts treated with 10 μg O_3_ showed a tendency (*p* = 0.06) to be more efficient than the other samples in the wound healing assay, thus accounting for the observed positive effects of oxygen–ozone therapy on wound healing [20,21].

The proliferation rate of LPS-activated fibroblasts was unaffected by the exposure to any gas, but a significantly lower proliferation was found in LPS-activated fibroblasts in comparison with the non-activated ones. This finding may be also related to the activated state of the cells: consistent with the results in the present investigation, a decreased proliferation rate (without an increase in the LDH release) has already been reported in lung fibroblasts submitted to LPS treatment [22,23].

As already recalled, fibroblasts are involved in wound healing and mediate the formation and remodeling of connective and epithelial tissues [24,25,26]. Migrating fibroblasts are motile cells characterized by superficial cellular protrusions, such as lamellipodia and filopodia [27]; in addition, filamentous projections are formed to remodel the collagen-rich extracellular matrix during wound healing [28]. Under our experimental conditions, surface protrusions were scarce in control and O_2_-treated non-activated fibroblasts, but they evidently increased after O_3_ exposure. It is worth noting that small local changes in the amount of reactive oxygen species (ROS), as induced by mild ozonation [15], may stimulate the polymerization of cytoskeletal actin [29,30,31] that is essential to form cell protrusions and promote adhesion [32,33]. However, the wound healing assay showed that the O_3_-induced increase in the surface processes was not paralleled by a higher migration rate, consistent with previous evidence that O_3_ exposure does not affect the cell migration capability [34]. 

On the other hand, O_3_ did not affect surface protrusions in LPS-activated fibroblasts, whose control samples showed similar amounts of these membrane processes as the O_3_-treated non-activated fibroblasts. This is consistent with the finding that cultured fibroblasts treated with LPS increase their ROS production [35,36,37], which in turn affects the organization of cytoskeletal proteins [29,30,31]. It can therefore be inferred that the low O_3_ concentrations tested in the present study are able to induce activation-like changes of the cell membrane in non-activated fibroblasts but do not affect already LPS-activated fibroblasts, thus avoiding their overstimulation and the possible scarring of the extracellular matrix [28]. 

It is known that the administration of low O_3_ concentrations is able to restore impaired Nrf2 pathways in many pathological conditions [38,39,40,41,42,43,44], thus inducing a cytoprotective response accounting for the therapeutic potential of O_3_. The mechanism relies on the stabilization of Nrf2 that mediates an antioxidant response by the Keap1/Nrf2 dependent pathway [5]: ozonation prevents Keap1-mediated degradation of Nrf2 and promotes its translocation to the nucleus [5,7]), where it activates the expression of ARE-driven genes [5,6,7,16]. This enables an efficient and rapid transcription of antioxidant genes without requiring a de novo synthesis of Nrf2. Accordingly, in the present study, the total amount of Nrf2 protein was unchanged in non-activated fibroblasts, while the expression of Hmox1 (i.e., the marker gene for the mild-O_3_-induced antioxidant response) increased in samples treated with 20 μg O_3_, similarly as it was observed in nervous cells [16]. Genes involved in oxidative stress responses were found to be upregulated also in primary periodontal ligament fibroblasts treated with O_3_ ultrafine bubble water [45]. In LPS-activated fibroblasts, the Nrf2 protein increased in 20 μg O_3_- and 30 μg O_3_-treated samples, probably due to the combined oxidative stress due to LPS and O_3_ exposure. Consistently, Hmox1 expression increased in a dose-dependent manner in O_3_-treated samples.

As stated above, fibroblasts are also involved in the regulation of the immune response (with complex and mutual interactions with the cells of the immune system) and are able to secrete different cytokines [46]. In particular, cultured fibroblasts secrete both IL-6 and TGF-β1 [47], as observed in our experimental model. Our data are also consistent with previous findings demonstrating that IL-6 secretion increases after LPS activation in vitro [48], while cell proliferation is inhibited in an autocrine pathway [22].

IL-6 exerts a pleiotropic effect on a broad spectrum of biological events and participates in the immune response as a potent pro-inflammatory cytokine involved in the acute inflammatory response; on the other hand, it also coordinates anti-inflammatory or repair-oriented activities essential for the resolution of inflammation [49]. In injured tissues, IL-6 is a major systemic alarm signal [50,51,52] involved in the activation of a variety of local and systemic host-defense mechanisms aimed at limiting tissue injury while stimulating angiogenesis, collagen production and organization, keratinocyte proliferation, and leukocyte infiltration [53,54,55,56]. The ability of low O_3_ concentrations to stimulate IL-6 secretion in fibroblasts is therefore compatible with the efficacy of O_2_–O_3_ therapy in wound healing and, more generally, in tissue repair [57,58,59,60]. In particular, low O_3_ concentrations seem to stimulate IL-6 secretion in LPS-activated fibroblasts more efficiently than in non-activated ones: this could be related to the activated state that makes the cells more responsive to stimuli. Interestingly, pre-treatment with low O_3_ concentrations proved to reduce IL-6 secretion in skin fibroblasts receiving doxorubicin, thus preventing the inflammatory effect of this potent cytotoxic drug [44]. Moreover, repeated and prolonged exposure of synovial fibroblasts isolated from patients affected by rheumatoid arthritis led to a decreased production of IL-6 [61]. The immunomodulation potential of O_3_ on fibroblasts therefore deserves detailed studies in view of targeted therapeutic approaches.

TGF-β1 also plays important roles as a key cytokine in the wound healing process, where it acts bidirectionally [62], promoting the synthesis of various extracellular matrix proteins [63,64,65,66,67] and potentiating angiogenesis [68,69,70], while inhibiting extracellular matrix degradation [71] and inflammatory response [72,73]. TGF-β1 has been also reported to enhance fibroblasts proliferation [74,75]. Therefore, its increased secretion in non-activated fibroblasts following 10 µg O_3_ treatment may be related to the higher proliferation rate found in this sample. On the other hand, LPS activation in vitro inhibits TGF-β1 production [48], consistently with the very low amount of TGF-β1 found in our LPS-activated fibroblasts. However, despite the inhibiting effect of LPS, 10 μg O_3_ proved to be capable of increasing TGF-β1 secretion also in LPS-activated fibroblasts; on the contrary, 20 μg O_3_ and 30 μg O_3_ treatments induce a higher stress and a stronger TGF-β1 inhibition, likely due to the activation of the Nrf2/ARE-mediated antioxidant signaling [76,77,78]. The stimulating effect of low O_3_ concentrations on TGF-β1 secretion observed in our in vitro model is consistent with the upregulation of this cytokine reported in cutaneous wounds undergoing accelerated repair following ozonated oil treatment [79,80]. 

## 4. Materials and Methods

### 4.1. Cell Culture and Treatment

Human lung fibroblasts (WI-26, ATCC) were chosen for the present study as a suitable in vitro model previously used to investigate the effects of drugs on extracellular matrix deposition [81] and remodeling [82] as well as the response to various stimuli [83,84,85]. The fibroblasts were grown in Dulbecco’s modified Eagle’s medium supplemented with 10% (*v*/*v*) fetal bovine serum, 1% (*w*/*v*) glutamine, 100 U of penicillin and 100 μg/mL streptomycin (all reagents were purchased from Gibco, Walthem, MA, USA) at 37 °C in a 5% CO_2_ humidified atmosphere.

The cells were treated with O_2_–O_3_ gas mixtures produced from medical-grade O_2_ by an OZO2 FUTURA apparatus (Alnitec, Cremosano, CR, Italy) that allows photometric real-time control of gas flow rate and O_3_ concentration. The concentrations of 10, 20, and 30 µg O_3_/mL O_2_ were chosen as these are usually administered in clinical practice and had been shown to be non-cytotoxic for different cultured cells [7,11,15,16]. The treatment with pure O_2_ was performed in order to discriminate the effect of O_3_ from O_2_ in the context of the O_2_–O_3_ gas mixtures. Controls consisted in cells submitted to the same handling but without exposure to gas.

The cells were trypsinized (0.25% trypsin in PBS containing 0.05% EDTA) (Gibco), when sub-confluent. For Western blot analysis and RT-qPCR, samples of 4 × 10^6^ cells were suspended in 10 mL medium into a 20 mL polypropylene syringe, then 10 mL of gas was added into the syringe using a sterile filter (Alnitec, Cremosano, CR, Italy) and the cell suspension was gently mixed with the gas for 10 min to allow the full reaction of cells with the gas [86]. For S-phase cells evaluation, SEM analysis, and wound healing assay, after trypsinization, the cells were seeded on glass slides placed in multi-well microplates, let to adhere for at least 24 h and then submitted to gas treatment as described in [87]. For LDH and cytokine assays, 2 × 10^4^ cells per 24-multi-well plate were seeded after gas treatment. At 24 h, the medium was collected and stored at −80 °C until analysis.

Some fibroblast samples were pre-incubated with 1 µg/mL LPS for 24 h as previously reported [23,88] in order to induce cell activation, and then processed as above.

### 4.2. Cytotoxicity

LDH, a cytosolic enzyme released by lysed cells, was evaluated as an estimate of the cytotoxic effect of gas exposure by using the CytoTox96 nonradioactive assay (Promega, Milan, MI, Italy). Cytotoxicity rate was estimated 24 h after the gas treatment in both non-activated and LPS-activated fibroblasts. Aliquots of medium were collected for each condition, placed in a 96-multi-well plate, mixed with the CytoTox 96 reagent and incubated for 30 min at room temperature. After addition of the stop solution, the absorbance was measured at 492 nm, and the data were corrected for culture medium background and normalized to the maximum LDH release (i.e., the one of lysed samples).

### 4.3. S-Phase Evaluation

The percentage of S-phase cells was assessed 24 h after treatment in both non-activated and LPS-activated fibroblasts, as a measure of the cell proliferation rate. The cells (2 × 10^4^ cells per 24 mm × 24 mm slides) were pulse-labelled with 20 μM BrdU (Sigma-Aldrich, St. Louis, MO, USA) for 30 min at 37 °C, then fixed with 70% ethanol and incubated for 20 min at room temperature in 2 N HCl to partially denature DNA; after neutralization with 0.1 M sodium tetraborate (pH 8.2) (Sigma-Aldrich) for 3 min, samples were washed in PBS, permeabilized for 15 min in PBS containing 0.1% bovine serum albumin and 0.05% Tween-20 (Sigma-Aldrich), and incubated for 1 h with a mouse monoclonal antibody recognizing BrdU (BD Diagnostics, Franklin Lakes, NJ, USA) diluted 1:20 in PBS. After two washes with PBS, samples were incubated for 1 h with an Alexa Fluor 488-conjugated anti-mouse secondary antibody (Molecular Probes, Invitrogen, Milan, MI, Italy), diluted 1:200. The cell samples were washed with PBS, stained for DNA with 0.1 μg/mL Hoechst 33342 (Abcam, Cambridge, United Kingdom) in PBS for 10 min, and finally mounted in PBS/glycerol (1:1). The percentage of BrdU-positive cells was evaluated in 30 randomly selected fields (40×) per experimental condition. For observation of all samples, an Olympus BX51 microscope (Olympus Italia S.r.l., Segrate, MI, Italy) equipped with a 100 W mercury lamp was used under the following conditions: 450–480 nm excitation filter (excf), 500 nm dichroic mirror (dm), and 515 nm barrier filter (bf) for Alexa Fluor 488; 330–385 nm excf, 400 nm dm, and 420 nm bf, for Hoechst 33342. Images were recorded with a QICAM Fast 1394 Digital Camera (QImaging, Surrey, BC, Canada) and processed with Image-Pro Plus software (Media Cybernetics, Inc., Rockville, MD, USA). 

### 4.4. Wound Healing Assay

For the wound healing assay, 20 × 10^4^ cells per well were seeded on 24 mm × 24 mm slides. After 24 h, when the cells were confluent, the cell monolayers were scratched with a sterile 200 µL pipette tip and immediately exposed to gas treatment. To evaluate cell migration, images at 4× magnification were taken at 0 h, 2 h, 6 h, and 24 h post-treatment using an inverted microscope (Leica DMIL, Leica Microsystems S.r.l., Buccinasco, MI, Italy) equipped with a camera (Optika Microscopes, Ponteranica, BG, Italy): the cell-free area was measured in a total of 12 randomly selected microscope fields per sample (4 fields in 3 independent experiments). The progressive reduction of the cell-free area was expressed as percentage, considering the value at time 0 as 100%.

### 4.5. Scanning Electron Microscopy

For SEM analysis, 2 × 10^4^ cells (both non-activated and LPS-activated fibroblasts) per well were seeded on round slides of 12 mm in diameter. After 24 h, the cell monolayers were gas exposed. At 24 h after the treatment, the cells were fixed with 2.5% glutaraldehyde in PBS for 2 h at 4 °C, washed in the same buffer, post-fixed with 1% OsO_4_ at 4 °C for 1 h and dehydrated with graded ethanol. The samples were then treated by a critical point dryer (CPD 030, BAL- TEC AG, Balzers, Liechtenstein), mounted on metallic specimen stubs and sputter-coated with gold (MED 010, BAL- TEC AG). SEM imaging was performed by an XL30 ESEM (FEI Italia S.r.l., Milan, Italy). Using ImageJ software (NIH), the length of cell surface facing the edge of the monolayers was measured both including and excluding cell protrusions in 20 cells per sample; the ratio between the two values was then calculated in order to obtain an index of cell surface irregularity (the higher the value, the rougher the cell). 

### 4.6. Western Blot Analysis

Non-activated and LPS-activated fibroblast samples were collected at 20 min post-gas-treatment and immediately frozen in liquid nitrogen to be then placed at −80 °C. Proteins were extracted according to standard procedures in RIPA buffer (150 mM NaCl, 10 mM Tris pH7.5, 1% NP40, 1% deoxycholate, 0.1% SDS) supplemented with phosphatase and protease inhibitors (Sigma-Aldrich).

Samples were resolved on Tris–glycine 4–20% gradient SDS-PAGE (BIO-RAD, Segrate, MI, Italy), blotted on PVDF membrane (BIO-RAD), and developed with ECL Western Blotting Substrate (Thermo Scientific, Rodano, MI, Italy). The following antibodies were used: anti-Nrf2 1:1000 (ab62532 Abcam) and Actin 1:5000 (ab8226 Abcam).

### 4.7. Real-Time Quantitative Polymerase Chain Reaction

RNA was extracted from non-activated and LPS-activated fibroblast samples after 24 h after the gas exposure by using the Qiagen RNeasy Plus mini kit (ref. 74134) (Qiagen S.r.l., Milan, Italy). cDNA was generated by SuperScript™ III Reverse Transcriptase (Invitrogen, cat. no. 18080093) (Thermo Fisher Scientific Inc., Waltham, MA, USA) and amplified at qPCR with Applied Biosystems™ SYBR™ Green PCR Master Mix (Applied Biosystems™ 4309155) (Thermo Fisher Scientific Inc.) using 2 distinct sets of primers specific for human Hmox1 (primers set 1: Forw: CCTAAACTTCAGAGGGGGCG, Rev: GACAGCTGCCACATTAGGGT; primers set 2: Forw: AGTCTTCGCCCCTGTCTACT, Rev: CTTCACATAGCGCTGCATGG). The Applied Biosystems Step-One Real-Time PCR System was used for amplification (Thermo Fisher Scientific Inc.). 

### 4.8. IL-6 and TGF-β1 Secretion 

The amount of IL-6 and TGF-β1 secreted was evaluated in the culture medium of both non-activated and LPS-activated fibroblasts 24 h after the gas treatment. For each sample, 4 × 10^5^ cells/mL were treated with gas; experiments were performed four times per sample and the medium was collected, centrifuged at 1500 g for 15 min, and the supernatants were finally stored at −80 °C. Quantitation of IL-6 and TGF-β1 was conducted on a Luminex Bio-Rad Bio-Plex 100 instrument (Bio-Rad Laboratories, Segrate, MI, Italy) coupled to the Bio-Plex Manager software, v6.0, which allows measuring multiple proteins in a single well. Briefly, 50 μL aliquots of undiluted cell medium were put in a 96-well plate (samples were run in duplicate). Superparamagnetic microspheres (beads) conjugated with fluorophores and antibodies against IL-6 and TGF-β were added to the assay wells. Incubation and washing steps were performed as per manufacturer’s recommendations, then the plate was loaded into the Luminex system for reading and signal quantitation.

### 4.9. Statistical Analysis

Data for each variable were presented as mean ± standard error (SE). Statistical comparison was performed by either the Kruskal–Wallis non-parametric test (cytotoxicity; wound healing assay, cytokines) followed by the Mann–Whitney test for pairwise comparison or the one-way analysis of variance (ANOVA) test (S-phase evaluation; index of cell surface irregularity, RT-qPCR) followed by Bonferroni’s post-hoc test. To RT-qPCR results, the test for linear trend was also applied. Statistical significance was set at *p* ≤ 0.05.

## 5. Conclusions

Taken together, the results of the present study not only extend to fibroblasts the notion that low O_3_ concentrations are safe for cells, but also provide original evidence that the administration of O_2_–O_3_ gas mixtures induces multiple effects on fibroblasts, depending on their activation state. In sum, in non-activated fibroblasts, O_3_ is able to stimulate proliferation, formation of cell surface protrusions, antioxidant response, and IL-6 and TGF-β1 secretion, while in LPS-activated fibroblasts, O_3_ stimulates antioxidant response and cytokines secretion without affecting cell proliferation and motility. It is therefore evident that the low O_3_ concentrations used in this study induce activation-like responses in non-activated fibroblasts, whereas, in fibroblasts already activated by LPS, the gas exposure potentiates the cell protective capability. Interestingly, most of the effects observed in non-activated fibroblasts are due to the exposure to 10 or 20 µg O_3_, i.e., the concentrations that have been already found as optimal for safely inducing positive response in various cell models [5,6,7,11,15,16]. This perfectly agrees with the low-dose concept in the medical use of O_3_ [89,90], which is increasingly applied in clinical practice.

The simple in vitro model used in the present study was a suitable tool to shed light on the specific responses of fibroblasts to low O_3_ concentrations; however, the observed effects should be investigated in a more complex network of mutual interactions of different cells and humoral factors, as it occurs in a living organism. Based on the present findings, further in vivo studies will elucidate the contribution of fibroblasts in the response to O_2_–O_3_ therapy, taking into account their multiple roles in tissue repair and homeostasis; this will provide novel information to properly modulate the O_3_ administration protocols for specific therapeutic needs.

## Data Availability

Not applicable.

## Supplementary Materials

The following are available online at https://www.mdpi.com/article/10.3390/ijms221810133/s1, Figure S1: Wound healing assay of non-activated fibroblasts, Figure S2: Wound healing assay of LPS-activated fibroblasts.
