# Supplementary material for: Low Ozone Concentrations Differentially Affect the Structural and Functional Features of Non-Activated and Activated Fibroblasts In Vitro
Source: Int J Mol Sci. 2021 Sep 20;22(18):10133. doi: 10.3390/ijms221810133 (PMC8466365; doi:10.3390/ijms221810133)
Supplement: Supplementary file 1 [file ijms-22-10133-s001.zip › Figure S2.pdf]

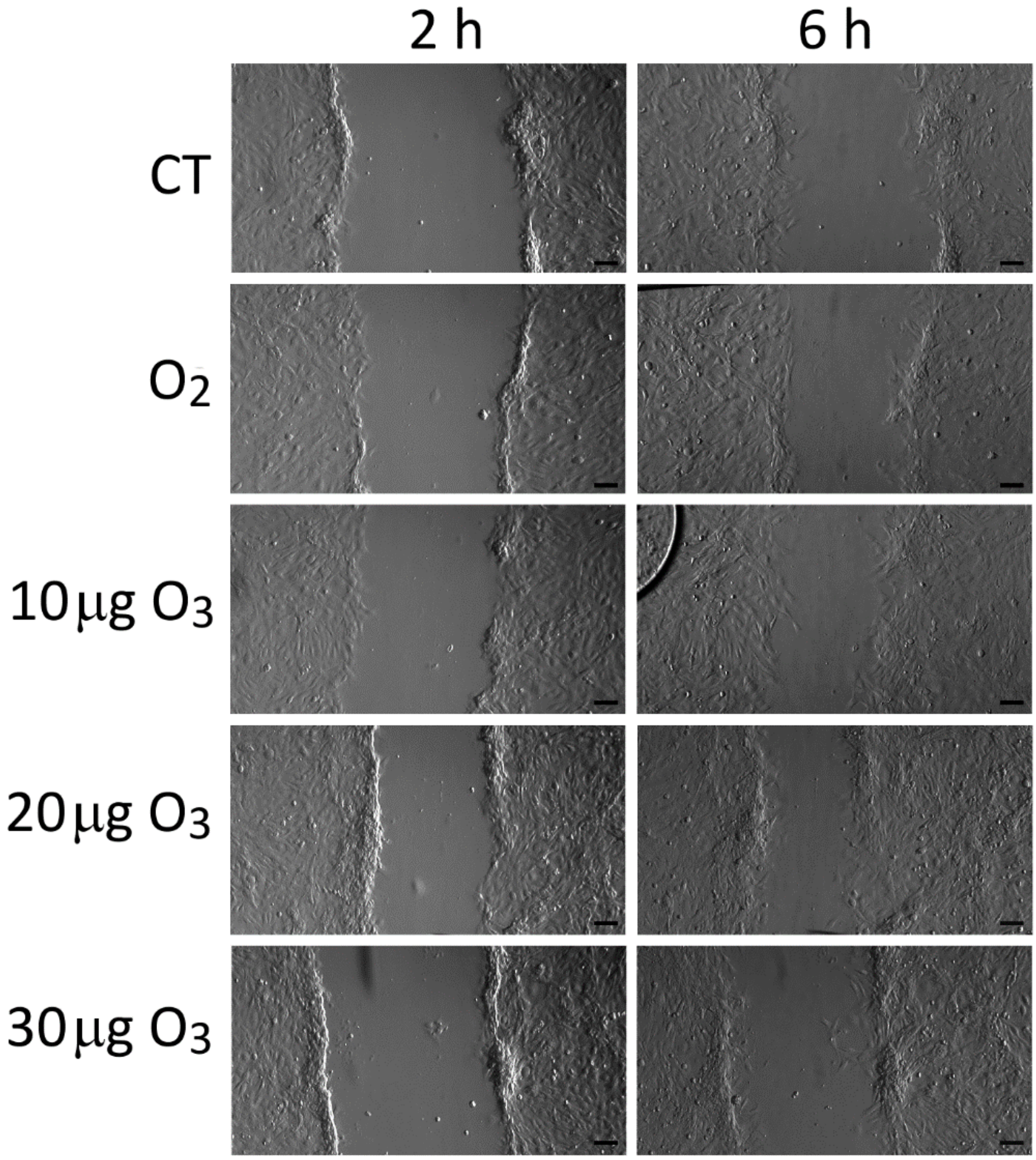

Figure S2. Wound healing assay of LPS-activated fibroblasts. Inverted microscope images of control (CT), O_2-, 10 µg O_3-, 20 µg O_3- and 30 µg O_3-treated fibroblasts at 2 h and 6 h. Bars: 200 µm.
